# Clinical, Radiological, and Histopathological Patterns of Allergic Fungal Sinusitis: A Single-Center Retrospective Study

**DOI:** 10.7759/cureus.9233

**Published:** 2020-07-17

**Authors:** Marzouqi A Salamah, Mazin Alsarraj, Nawaf Alsolami, Kamal Hanbazazah, Abdulmajeed M Alharbi, Wael Khalifah

**Affiliations:** 1 Otolaryngology-Head and Neck Surgery, Ohud Hospital, Al-Madinah Al-Munawarah, SAU; 2 Otolaryngology-Head and Neck Surgery, King Fahad General Hospital, Jeddah, SAU; 3 Otolaryngology-Head and Neck Surgery, King Fahad Armed Forces Hospital, Jeddah, SAU; 4 Otolaryngology-Head and Neck Surgery, King Abdulaziz University Hospital, Jeddah, SAU; 5 Otolaryngology-Head and Neck Surgery, Jeddah University, Jeddah, SAU

**Keywords:** allergic, eosinophilic, fungal sinusitis, imaging, histology, mucin

## Abstract

Objectives

To explore the clinical, pathological, and imaging characteristics of allergic fungal sinusitis (AFS) and to analyze the correlation of disease duration with imaging and histopathology findings.

Methods

We reviewed all cases of AFS managed at the otorhinolaryngology department of King Fahad Armed Forces Hospital, Jeddah, Saudi Arabia. Demographic and clinical features were collected, as well as imaging and histopathological findings, which were analyzed by time from onset.

Results

Forty-six patients were diagnosed AFS, representing 11.8% of total sinusitis cases; 25 (54.3%) were female, with mean (SD) age=33.57 (11.76). Patients presented with multiple symptoms of chronic rhinosinusitis (43, 93.5%), chronic headache (14, 30.4%), and hyposmia (2, 4.3%), and 36 (78.3%) were diagnosed late (≥5 years after onset). AFS involved all four sinuses in 32 (69.6%) patients and was bilateral in >53.5% of infected sinuses. Imaging showed increased intrasinus attenuation (88.2%-95.3%), complete opacification (74.4%-85.3%), sinus expansion (35.3%-51.2%), remodeling (20.6%-37.2%), wall thinning (41.2%-58.1%), and involvement of adjacent soft tissue (11.8%-25.6%), depending on the sinus type. Histology evidenced eosinophilic mucin (45.7%), eosinophils (91.3%), fungal hyphae (93.5%), and Charcot-Leyden crystals (6.5%). Patients who were diagnosed late had a higher percentage of imaging and pathological lesions, principally, the expansion and wall thinning of involved sinuses (p<0.050).

Conclusion

AFS represents a significant proportion of chronic sinusitis cases treated in the otorhinolaryngology department and is often diagnosed late with extensive forms. Major efforts should be made to improve the early diagnosis and management of such disease, including raising awareness about this entity among general practitioners and family physicians to enhance clinical suspicion and detection rate.

## Introduction

Allergic fungal sinusitis (AFS), or eosinophilic fungal rhinosinusitis (EFRS), is a barely recognized pathologic entity that belongs to the fungal rhinosinusitis group [1]. It is broadly defined as a non-invasive fungal infection of sinuses inducing a marked type I hypersensitivity reaction that overshadows the clinical picture [2-3]. It is characterized by pathognomonic eosinophilic mucin-containing hyphae besides other distinctive histological and imaging features that contribute to the diagnosis [4-7].

We estimated the prevalence and explored the clinical, pathological, and imaging characteristics of AFS cases managed in our institution. We also analyzed the evolution over time of imaging and pathological findings and their correlation with hypereosinophilia.

## Materials and methods

This was a retrospective review of all cases of AFS that were diagnosed and treated at the otorhinolaryngology department of King Fahad Armed Forces Hospital (KFAFH), Jeddah, Saudi Arabia, between January 2009 and January 2019. Inclusion criteria were applied for patients with histologically confirmed non-invasive fungal sinusitis (IFS), which was carried out following signs suggestive of AFS on radiology (computed tomography (CT) and magnetic resonance imaging (MRI)). Histological diagnosis was confirmed upon direct isolation of fungi microorganisms or the presence of fungal hyphae with eosinophilic mucin in the submucosa, blood vessels, and bone. The study excluded patients with other types of fungal sinusitis, including invasive fungal types, as well as patients with non-fungal sinusitis. The study was approved by the institutional review board of KFAFH.

Mucus and inflamed tissues were completely removed from all patients by functional endoscopic sinus surgery, with optional corticosteroid therapy by an intranasal or systemic route. Surgical specimens were examined intraoperatively to confirm the presence of eosinophilic mucin, identified as a firm and highly glutinous substance with a "peanut butter-like" color, which is highly suggestive of fungal sinusitis [7-8]. Subsequently, samples were collected in sterile normal saline bottles and referred to the histopathology and microbiology laboratories. Histologic examination was done by staining with Gomori's methenamine silver (GMS) and periodic acid-Schiff (PAS) to identify fungal structures. Fungal cultures were done by standard methods on Sabouraud's agar media [7].

The following data were collected: 1) demographic data, including age and gender; 2) clinical presentation, including time from onset, presenting symptoms, and the number of sinuses involved; 3) imaging findings, including sinus involved (sphenoid, maxillary, frontal, and/or ethmoid), side (right, left, or bilateral), presence of mucosal thickening, increased intrasinus attenuation, complete opacification, sinus expansion, remodeling, or wall thinning and the involvement of adjacent soft tissues in each involved sinus [9-10]; 4) laboratory data, including blood eosinophil rate, and pathology findings, including the presence of eosinophilic mucin, eosinophils, fungal hyphae, and Charcot-Leyden crystals; and 5) management, including therapeutic strategy and postoperative medication (antihistaminic agents, antifungal drops, corticosteroids, and antibiotics).

Statistical methods

Statistical analysis was performed with the Statistical Package for Social Sciences version 21.0 for Windows (IBM Corp., Armonk, NY). The prevalence of AFS was calculated as the percentage of patients diagnosed and treated as AFS among all cases of chronic rhinosinusitis treated in the same period; the result was presented as a percentage with a 95% confidence interval (95% CI). Descriptive statistics were used to present the demographic and clinical characteristics, as well as the imaging and pathological characteristics. Imaging findings were described separately for sphenoid, frontal, maxillary, and ethmoid sinuses. Categorical variables are presented as frequency and percentage, while continuous variables are presented as mean ± standard deviation (SD). Evolution over time of the imaging and pathological findings was analyzed by comparing patients with short disease duration (time from onset <5 years) with those having five to 10 years and a longer disease duration; the analysis used the chi-square test or Fisher’s exact test, as appropriate. Further, the association of hypereosinophilia with demographic, clinical, and histopathological factors was analyzed using the chi-square test or Fisher’s exact test, as appropriate. A p-value of <0.05 was considered to reject the null hypothesis.

## Results

Patients’ demographic and clinical characteristics

Forty-six patients were diagnosed with AFS in the institution, representing 11.8% of 390 cases of sinusitis treated in the same period. The demographics of the 46 cases showed that 25 (54.3%) were female, mean (SD) age=33.57 (11.76) years, with 54.4% being above 30 years. On presentation, 43 (93.5%) patients presented with multiple symptoms of chronic rhinosinusitis. Notably, 14 (30.4%) patients complained of a chronic headache and two (4.3%) of hyposmia. In medical history, we noted hypertension (5, 10.9%), asthma (5, 10.9%), and diabetes (4, 8.7%), while seven (15.2%) had a history of sinus surgery. In the majority of cases, the diagnosis was made late, i.e., after 5+ years of symptom onset (36, 78.3%) and had four sinuses involved (32, 69.6%) (Table 1).

**Table 1:** Participants’ demographic and clinical characteristics (N=46) Values are frequencies and percentages, except if otherwise specified. § Other medical history included tympanoplasty (1 case), decreased vision (1), hypothyroidism (1), hepatitis B virus infection (1), G6PD deficit (1), end-stage renal disease (1), epilepsy (1), eczema & food allergy (1); SD: standard deviation; G6PD: glucose-6-phosphate dehydrogenase

Parameter	Category	Frequency	Percentage
Demographics			
Gender	Male	21	45.7
	Female	25	54.3
Age	Mean, SD	33.57	11.76
	Up to 20	7	15.2
	21-30	14	30.4
	31-40	12	26.1
	>40	13	28.3
Medical history			
Comorbidities	Asthma	5	10.9
	Diabetes	4	8.7
	Hypertension	5	10.9
	Other^§^	8	17.4
Past sinus surgery	No	39	84.8
	Yes	7	15.2
Clinical picture			
Time from onset (years)	Mean, SD	7.57	3.19
	Median, interquartile	9.00	5.00
	<5	10	21.7
	5-<10	18	39.1
	10+	18	39.1
Presenting symptoms	Multiple symptoms of chronic rhinosinusitis	43	93.5
	Chronic headache	14	30.4
	Hyposmia	2	4.3
	Fever	0	0.0
	Polyp (endoscopy)	46	100.0
No. sinuses involved	1	3	6.5
	2	4	8.7
	3	7	15.2
	4	32	69.6

Imaging findings

Among all patients, fungal infection involved the sphenoid (40, 87.0%), maxillary (43, 93.5%), ethmoid (43, 93.5%) and frontal (34, 73.9%) sinuses and was bilateral in 53.5% to 58.8% of all infected sinuses. All infected sinuses presented mucosal thickening (100.0%), and slight variations across the given sinus were observed regarding other CT imaging findings, including increased intrasinus attenuation (88.2%-95.3%), complete opacification (74.4%-85.3%), sinus expansion (35.3%-51.2%), remodeling (20.6%-37.2%), wall thinning (41.2%-58.1%), and involvement of adjacent soft tissue, including the medial orbital wall (11.8%-25.6%).

Hypereosinophilia and histological findings

Hypereosinophilia was found in 31 (67.4%) patients, with no difference across gender (p=0.243), age category (p=0.948), or the number of sinuses involved (p=0.217); however, it was relatively more frequent in patients diagnosed at a relatively early stage, i.e. < five years from onset (80.0%), compared to their counterpart (61.1% -66.7%), with no statistical significance (p=0.591). Pathologically, eosinophilic mucin was present in 45.7% of patients and was inversely associated with hypereosinophilia (p=0.047). Further, pathology was marked by the presence of eosinophils (91.3%), fungal hyphae (93.5%), Charcot-Leyden crystals (6.5%), and other inflammatory cells (47.8%) (Table 2 and Table 3).

**Table 2:** Imaging findings of allergic fungal sinusitis (N=46) § Of at least one side of the involved sinus; %t: percentage out of the total (N=46); %s: percentage out of the given sinus

Parameter	Sinus											
	Sphenoid			Maxillary			Frontal			Ethmoid		
	N	%t	%s	N	%t	%s	N	%t	%s	N	%t	%s
Involvement	40	87.0	100.0	43	93.5	100.0	34	73.9	100.0	43	93.5	100.0
Side												
Right	11	23.9	27.5	10	21.7	23.3	5	10.9	14.7	7	15.2	16.3
Left	7	15.2	17.5	10	21.7	23.3	9	19.6	26.5	11	23.9	25.6
Bilateral	22	47.8	55.0	23	50.0	53.5	20	43.5	58.8	25	54.3	58.1
Mucosal thickening	40	87.0	100.0	43	93.5	100.0	33	71.7	97.1	43	93.5	100.0
Increased intrasinus attenuation	38	82.6	95.0	41	89.1	95.3	30	65.2	88.2	39	84.8	90.7
Complete opacification ^§^	34	73.9	85.0	34	73.9	79.1	29	63.0	85.3	32	69.6	74.4
Expansion	19	41.3	47.5	17	37.0	39.5	12	26.1	35.3	22	47.8	51.2
Remodelling	13	28.3	32.5	9	19.6	20.9	7	15.2	20.6	16	34.8	37.2
Wall thinning	22	47.0	55.0	20	43.5	46.5	14	30.4	41.2	25	54.3	58.1
Involvement of adjacent soft tissue	8	17.4	20.0	7	15.2	16.3	4	8.7	11.8	11	23.9	25.6

**Table 3:** Biological and histopathological findings in fungal sinusitis (N=46)

Parameter	Level	Frequency	Percentage
Biology			
Eosinophils rate (%)	Normal (0-4)	15	32.6
	High (>4)	31	67.4
Histopathology			
Eosinophilic (allergic) mucin	Absence	25	54.3
	Presence	21	45.7
Eosinophils	Absence	4	8.7
	Presence	42	91.3
Fungal hyphae	Absence	3	6.5
	Presence	43	93.5
Charcot-Leyden crystals	Absence	43	93.5
	Presence	3	6.5
Other inflammatory cells	Absence	24	52.2
	Presence	22	47.8
	Neutrophils	12	26.1
	Lymphocytes	14	30.4
	Plasma cells	13	28.3
	Mononuclear cells	1	2.2

Management

**Figure 1:**
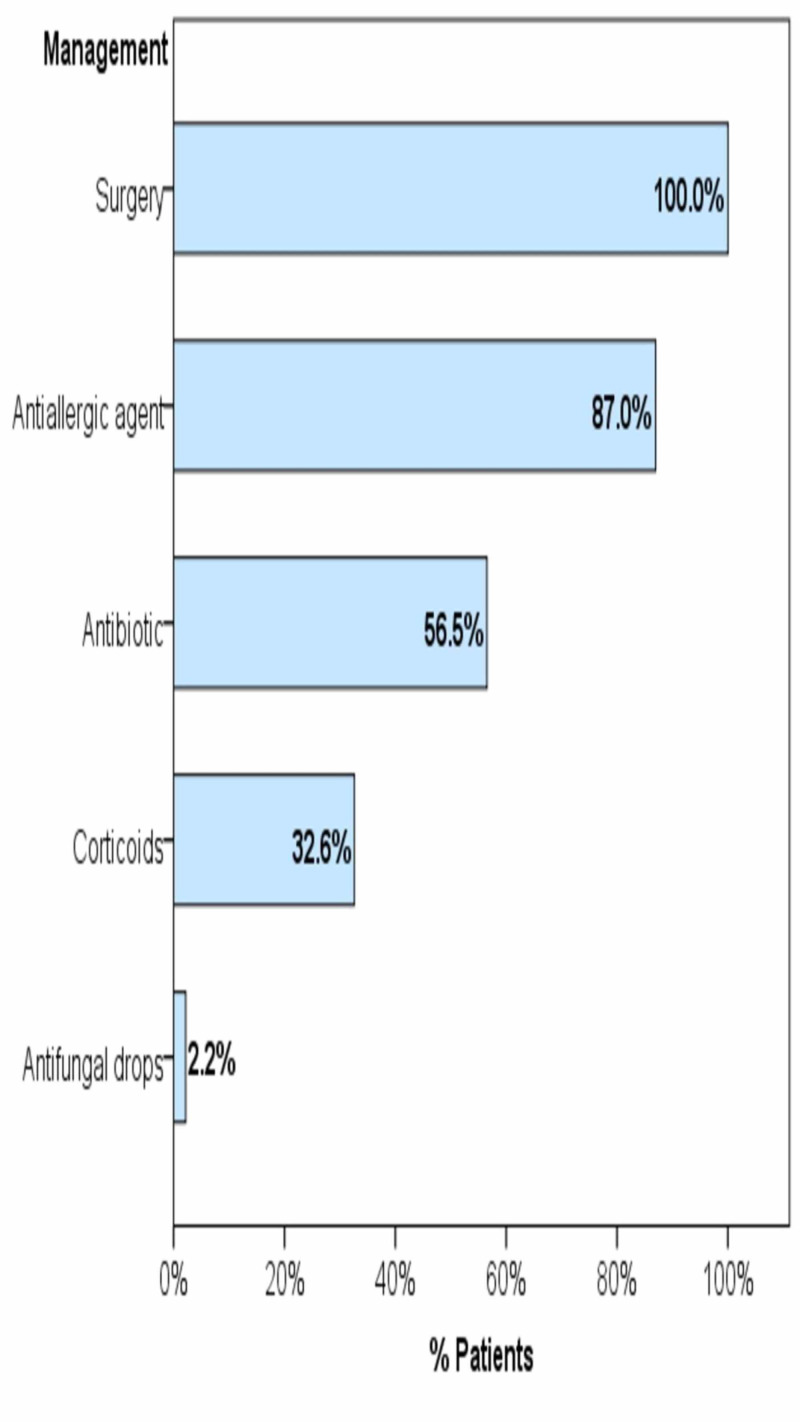
Management strategies of the cases of fungal sinusitis Bars represent the percentage of patients who have received the given treatment

All patients underwent surgery (46, 100.0%). Prior to diagnosis, pharmaceutical prescriptions included antiallergic agents (40, 87.0%), antibiotics (26, 56.5%), and corticosteroids (15, 32.6%); whereas antifungal drops were prescribed for only one patient (Figure 1).

Evolution over time of imaging and histopathology

At an earlier stage (time from onset <5 years), sinus expansion and wall thinning are less commonly found in involved sinuses (30.0% and 20.0%, respectively) than at a later stage (>55.6% and >72.2%, respectively); both associations are statistically significant (p=0.018 and 0.006, respectively). Besides, patients with longer disease duration (time from onset ≥5 years) are likely to have more frequent involvement of their four sinuses, with more frequent remodeling and involvement of adjacent tissue; however, these results were not statistically significant. Likewise, although not statistically significant, eosinophilic mucin and other inflammatory cells tend to be more frequent in patients with longer disease duration, while Charcot-Leyden crystals tend to be less frequent over time (Table 4 and Table 5).

**Table 4:** Factors associated with hypereosinophilia Test used: F, Fisher’s exact test; otherwise, chi-square test. * statistically significant result (p<0.05)

	Hypereosinophilia		p-value
	N	%	
Demographic factors				
Gender	Male	16	76.2	
	Female	15	60.0	.243
Age	Up to 20	5	71.4	
	21-30	10	71.4	
	31-40	8	66.7	
	>40	8	61.5	.948
Clinical factors				
Time from onset (years)	<5	8	80.0	
	5-<10	11	61.1	
	10+	12	66.7	.591
No. sinuses involved	1	1	33.3	
	2	3	75.0	
	3	3	42.9	
	4	24	75.0	.217
Histopathological factors				
Eosinophilic mucin	Absence	20	80.0	
	Presence	11	52.4	.047*
Eosinophils	Absence	2	50.0	
	Presence	29	69.0	.587^F^
Fungal hyphae	Absence	2	66.7	
	Presence	29	67.4	1.000^ F^
Charcot-Leyden Crystals	Absence	29	67.4	
	Presence	2	66.7	1.000^ F^
Other inflammatory cells	Absence	18	75.0	
	Presence	13	59.1	.250

**Table 5:** Over time evolution of imaging and histopathology findings (N=46) § Of at least one side; * statistically significant difference; test used: chi-square test

Time from onset, years						p-value
<5 (N=10)		5-<10 (N=18)		10+ (N=18)		
N	%	N	%	N	%	
Imaging							
No. Sinuses involved							
1	2	20.0	0	0.0	1	5.6	
2	0	0.0	0	0.0	4	22.2	
3	2	20.0	3	16.7	2	11.1	
4	6	60.0	15	83.3	11	61.1	.077
Mucosal thickening	10	100.0	18	100.0	18	100.0	-
Increased intrasinus attenuation	9	90.0	18	100.0	17	94.4	.438
Complete opacification ^§^	8	80.0	17	94.4	16	88.9	.500
Expansion	3	30.0	15	83.3	10	55.6	.018*
Remodeling	2	20.0	11	61.1	7	38.9	.097
Wall thinning	2	20.0	14	77.8	13	72.2	.006*
Involvement of adjacent soft tissue	1	10.0	9	50.0	5	27.8	.082
Histopathology							
Eosinophilic mucin	2	20.0	9	50.0	10	55.6	.174
Eosinophils	9	90.0	17	94.4	16	88.9	.828
Fungal hyphae	9	90.0	18	100.0	16	88.9	.354
Charcot-Leyden crystals	2	20.0	1	5.6	0	0.0	.119
Other inflammatory cells	3	30.0	8	44.4	11	61.1	.269

## Discussion

Summary of findings

In this single-center, retrospective chart review, AFS is found in approximately 11.8% of patients who presented at otorhinolaryngology for chronic rhinosinusitis. Patients with AFS are often diagnosed late, at an advanced disease stage, and present with atypical symptomatology. CT imaging revealed a high percentage of multiple and bilateral sinus involvement, with a considerable ratio of bone and tissue damage, including remodeling, wall thinning, and the involvement of adjacent soft tissue that increases with disease duration. Pathology was dominated by the presence of eosinophils and fungal hypha, while eosinophilic mucin was present in approximately half of the patients.

Case definition of AFS

The definition and diagnostic criteria of AFS are still under debate, and minor progress has been made in the last two decades to achieve a consensus [11]. Historically, the concept of the combined allergic and fungal pathological processes in sinuses was first described in 1976 by Safirstein, who reported the case of allergic bronchopulmonary aspergillosis simultaneously involving the patient’s sinuses [12]. Subsequently, several similar cases were reported and different lists of criteria were suggested based on each author’s clinical experience and the available literature then. These criteria included the characteristic eosinophilic-mucin containing hyphae, along with a positive fungal strain or culture, in the absence of tissue invasion by fungi, in addition to other suggestive (or supportive) clinical and biological evidence of an allergy such as positive atopic history, nasal polyposis, absence of immunodeficiency, and elevation of total or specific immunoglobulin E (IgE) or a positive skin test to fungal antigens [5,13-15]. However, these diagnostic criteria were not constantly reported in the literature, and the clinical picture overlapped with other entities of non-allergic fungal sinusitis in a considerable number of reported cases [16]. This is consistent with our study showing the presence of sinus polyposis on endoscopic examination, in 100% of cases, while characteristic eosinophilic mucin was present in less than half. On the other hand, the strict application of the aforementioned criteria may lead to several AFS cases going undiagnosed.

More recently, while several researchers debated the role of fungi in the genesis of AFS [17-21], others attempted to establish the immunologic difference between AFS and other types of chronic rhinosinusitis by highlighting the major role of allergy evidenced by significantly higher levels of allergy markers, such as total IgE, IgG anti-Alternaria-specific antibodies (UniCAP 100), and IgE antifungal antibodies in the sera of patients with AFS [22]. In the present study, the blood eosinophils rate was the only biological marker of allergy that was available in patients’ files, which was found to be elevated in two-thirds of the patients (67.3%), and was relatively more frequent in patients diagnosed at an early stage (80.0%).

A varying prevalence

Lack of consensus on AFS case definition, along with the relative rarity of the disease, further resulted in a fluctuating epidemiological picture, with a prevalence ranging between 5% and 27% of refractory chronic rhinosinusitis cases [23], 6% and 9% of rhinosinusitis cases requiring surgery [14], and 9% and 12% in sinonasal polyposis [24-25]. The highest figures were reported in India, where the prevalence of AFS was estimated to be between 56% and 79% of all cases of chronic rhinosinusitis [26-29]. Comparably, in the present study, AFS cases represented 11.2% of the total cases of chronic sinusitis that required surgical treatment.

Highly presumptive clinical and radiological signs

In routine practice, the diagnosis of AFS is suspected on a set of clinical and radiological arguments, then confirmed by an intraoperative examination of mucin and pathological and microbiological examinations postoperatively [1-11]. The present study highlighted several clinical signs that should lead the physician to suspect AFS. Together, these signs outline a clinical picture of chronic, non-febrile rhinosinusitis involving multiple sinuses and developing for several years, which does not respond to standard treatment. Classically, the literature reports the young age of the patient and te=he absence of pain as additional alerting signs [30], which is consistent with the young population (mean age ~34 years) and the low percentage of headache (~30%) in the present study. Radiologically, mucosal thickening was constantly found, and involved sinuses were completely opacified with increased intrasinus attenuation in majority cases, while signs of tissue and bone damage were less frequent and likely to be associated with advanced stage. In agreement with these findings, the literature exposes a set of radiological criteria, including typical opacifications with central hyper-attenuation in a CT scan, with the highlight of multiple sinus involvement. Additionally, eosinophilic mucin is characteristically identified by central low T1 and T2 void, as a result of its high protein concentration and low free water and mineral content in mucin. Besides, bone damage, including skull base erosion and/or orbital erosion, are reported to be relatively frequent (up to 56%) and are considered distinctive of AFS from non AFS [11].

Promoting specialist referral to enhance early detection

Acknowledgment of the aforementioned clinical and radiological arguments should systematically lead to the referral of the patient to an otorhinolaryngology specialist. However, to effectively enhance early detection, specialist referral should be encouraged for any case of chronic rhinosinusitis in atopic individuals, with the absence of bacterial infection signs and failure to respond to conventional treatments. This simple take-home message should be disseminated among general practitioners (GPs) and family physicians, as well as the general population, thereby enabling timely and appropriate management by the specialist to reduce the risk of irreversible damage.

Towards a new diagnostic approach

While no consensus could be reached regarding the diagnostic criteria, a comprehensive approach based on the distinctive pathophysiological features might be more relevant to address the AFS diagnosis. According to the updated classifications, AFS is described as a fundamentally immunoallergic pathologic process that is triggered and eventually sustained by fungi colonization. That is, the main symptomatology is explained by an allergy-mediated inflammatory process, and this could be considered as the Comprehensive Criterion number one (CC1) in the proposed diagnostic approach of AFS. Comprehensive Criterion number two (CC2) would theoretically be the presence of fungi, which can be evidenced by pre- or postoperative mycological examination of the sinus and nasal mucus. On the other hand, the non-invasiveness of the mucous membrane by fungi (CC3) constitutes the frontier that separates AFS from invasive forms of fungal sinusitis, which is a genuine chronic fungal infection commonly observed in immunosuppressed patients [11]. Based on this paradigm, the proposed new diagnostic approach assumes that each of these three comprehensive criteria could be fulfilled by relevant clinical, biological, radiological and pathological, and microbiological signs, the combination of which may differ from a patient to another.

Limitations

The main limitation of this study is the lack of relevant clinical and biological data such as the immune status of the patients, the history of nasal polyposis, total and fungal-specific IgE, and so on, which are mainly imputable to the retrospective design. Furthermore, follow-up data, such as success rate, recurrence rate, time to recurrence, etc., were missing and could provide valuable insight into treatment efficacy.

## Conclusions

AFS patients represent a significant percentage among cases of chronic sinusitis treated in otorhinolaryngology and are often diagnosed late, with extensive forms. Major efforts should be made to enhance the early diagnosis and management of such disease, in order to improve the outcome and reduce the risk of irreversible damage. Such measures include raising awareness about this entity among general practitioners and family physicians to enhance clinical suspicion and encourage referral to specialists of any case of refractory chronic rhinosinusitis. Given the continuous controversy regarding the definition and diagnosis criteria of AFS, we proposed a new diagnostic approach, which assumes an immunoallergic component, the presence of fungi, and the absence of mucous membrane invasion by fungi as comprehensive criteria that may be fulfilled by relevant clinical, biological, radiological and pathological, and microbiological signs; the combination of which may differ from one patient to another.
